# The e-BRAVE study: A prospective web-based cohort and biobank of women carriers of BRCA mutations

**DOI:** 10.1177/03008916251353420

**Published:** 2025-08-09

**Authors:** Andreina Oliverio, Carlotta Meli, Eleonora Bruno, Michela Bianchi, Giada Sassi, Elisabetta Venturelli, Ambra Cesareo, Claudio Pighini, Margherita Patruno, Maria Di Gennaro, Stefania Tommasi, Antonella Daniele, Silvia Schiavone, Letizia Galasso, Stefano Magno, Gianluca Franceschini, Alberta Ferrari, Robert Fruscio, Daniele Morelli, Claudia Chiodoni, Siranoush Manoukian, Patrizia Pasanisi

**Affiliations:** 1 Nutrition Research and Metabolomics Unit, Department of Experimental Oncology, Fondazione IRCCS Istituto Nazionale dei Tumori di Milano, Milano, Italy; 2 Hereditary Digestive Tract Tumors Unit, Department of Oncologycal Surgery, Fondazione IRCCS Istituto Nazionale dei Tumori di Milano, Milano, Italy; 3 LifeChargeR srl, Milano, Italy; 4 Department of Electronics, Information, and Bioengineering, Politecnico di Milano, Milano, Italy; 5 Center for Study of Heredo-Familial Tumors, IRCCS Istituto Tumori “Giovanni Paolo II”, Bari, Italy; 6 Molecular Diagnostics and Pharmacogenetics Unit, IRCCS Istituto Tumori “Giovanni Paolo II”, Bari, Italy; 7 Pathology Anatomy- Clinical Nutrition Unit, IRCCS Istituto Tumori “Giovanni Paolo II”, Bari, Italy; 8 Department of Biomedical Sciences for Health, University of Milan, Milano, Italy; 9 Multidisciplinary Breast Center, Fondazione Policlinico Universitario A. Gemelli IRCCS, Roma, Italy; 10 Hereditary Breast and Ovarian Cancer Unit and General Surgery 3-Senology, Surgical Department, Fondazione IRCCS Policlinico San Matteo, and University of Pavia, Pavia, Italy; 11 Gynecology Unit, Department of Medicine and Surgery, University of Milan-Bicocca, IRCCS San Gerardo dei Tintori, Monza, Italy; 12 Department of Medicine and Surgery, University of Milan-Bicocca, Milan, Italy; 13 Department of Advanced Diagnostic Services, Fondazione IRCCS Istituto Nazionale dei Tumori di Milano, Milano, Italy; 14 Molecular Immunology Unit, Department of Experimental Oncology, Fondazione IRCCS Istituto Nazionale dei Tumori di Milano, Milano, Italy; 15 Medical Genetics Unit, Department of Medical Oncology and Hematology, Fondazione IRCCS Istituto Nazionale dei Tumori di Milano, Milano, Italy

**Keywords:** web-based cohort, BRCA, prevention, lifestyle, gene- environment interaction

## Abstract

**Background:**

Women carriers of *BRCA1/2* mutations face a very high lifetime risk (penetrance) of developing breast and/or ovarian cancer. A sizeable proportion of carriers, however, does not develop cancer at all or develop it only late in life, thus suggesting a potential modulation of this risk. Epidemiological studies have suggested that other genetic (polymorphisms) and environmental factors (lifestyle) affect penetrance. However, data regarding these associations mainly come from retrospective case-control analyses and the results are likely to be distorted by bias.

**Aims:**

The e-BRAVE (Brca, ReseArch, Virtual, Education) study aims to create a web-based prospective cohort and biological bank of unaffected women carriers of *BRCA1/2* mutations to investigate the role of polymorphisms and environmental factors, and their interaction, in the occurrence of primary BRCA-related cancers.

**Methods:**

An innovative digital platform (including a mobile App) will be used to empower the synergy between participants and researchers, supporting engagement with women, adherence to intervention plan, self-empowerment, flanked by activities tracking and monitoring.

**Results:**

Based on the incidence data in previous studies, we estimate to observe an overall incidence of ~3.7% year.

**Conclusion:**

The success of this study will ensure the definition of further predictive risk models and comprehensive recommendations aimed at improving management and health of BRCA women.

## Introduction

*BRCA1/2* genes pathogenic variants (mutations) have an estimated incidence of about 1 case per 400 people, and are responsible, in Italy, for about 4000 new cases/year of breast cancer (BC) and 800 of ovarian cancer (OC).^1^ Due to their high lifetime risk (penetrance) of developing BC (~ 55% compared to 12% in the general population) and/or OC,^2–5^ women carriers attend life-long health services, and intensive programs of surveillance and prophylactic surgery. A sizeable proportion of carriers, however, do not develop cancer at all. This incomplete penetrance suggests that other factors influence the risk, genetic (polymorphisms)^6^ and/or “environmental” (lifestyle and metabolic/hormonal/inflammatory factors).^7,8^

The cancer risk is higher if genotype carriers are obese and gain weight during life^9–11^ especially for post-menopausal BC^12,13^ and for OC.^14^ Higher fat mass and low serum levels of adiponectin are associated with BRCA-related cancers, especially in women carriers of *BRCA1* mutations.^15,16^ High serum levels of insulin-like growth factor-I (IGF-I) are associated with an increased penetrance.^17^ Physical activity (PA) reduces BC risk in women with BRCA mutations.^18–22^ PA raises adiponectin and lowers leptin,^22^ and decreases inflammation and oxidative stress^23,24^ promoting a better regulation of insulin.^25^ Several genome-wide association studies identified single nucleotide polymorphisms (SNPs) associated with the levels of metabolic and inflammatory parameters as IGF-I,^26,27^ adiponectin,^28,29^ and insulin,^30,31^ suggesting that their plasma levels are under complex genetic control.

*BRCA1*/*2* genes are involved in the restoration of the original DNA sequence at the damage site. MicroRNAs (miRNAs), a class of short non-coding RNAs, regulate, at transcriptional and post-transcriptional levels, the DNA damage sensor, signal transducer and effector genes in cancer cells. Our group found that miR-17, miR-21, and let-7a were significantly over-expressed in familial compared to sporadic BC, while miR-124 was significantly underexpressed.^32^ In our previous trial, five tumor-suppressor miRNAs were significantly up-regulated after six months of Mediterranean diet (MedDiet).^33^ However, the knowledge about miRNAs expression and BRCA-related cancers is still poor.

Gut microbiota is able to modify and produce metabolites with local and systemic effects. The short-chain fatty acids (SCFAs) produced from dietary fiber digestion serve as energy source for colonocytes, incise in the regulation of inflammation, and modulate proliferation, metabolism, and immune system of tumor cells.^34,35^ MedDiet promotes SCFAs production, particularly butyrate.^36^ Conversely, western dietary pattern is associated with bacterial dysbiosis,^37^ local inflammation and changes in intestinal permeability, that facilitate the passage of the intestinal contents into the systemic organism, thus influencing the chronic inflammatory state. The lipopolysaccharides (LPS) of bacterial origin has been hypothesized to potentiate chronic low-grade inflammation.^38,39^ There is a single study on the microbiota in patients with BRCA mutation focused on the relationship between fecal SCFA and intestinal permeability, but it did not investigate the association with BRCA-related cancers.^40^

Overall, all the epidemiological data regarding the association of the “environmental” penetrance modulators and BRCA*-*related cancers mainly come from retrospective case-control analyses in carriers and the results are likely to be distorted by bias.

In order to assess the specific role of the modulators involved in BRCA penetrance, large prospective studies, with precisely measured exposures and accurately assessed potential confounders, are needed. Internet-based epidemiological studies are emerging, especially as regards the recruitment of large and geographically dispersed populations, the ability to incorporate novel exposure assessment tools based on computer technology and the rapidly expanding access across social strata. Evidence from literature supports the effectiveness of via-web studies.^41–44^ Thanks to the Internet, it is possible to gradually collect huge amounts of data from a large sample of volunteers that can be automatically verified and processed. In this context we have designed the e-BRAVE (**B**rca, **R**ese**A**rch, **V**irtual, **E**ducation) study, a web-based prospective cohort and biological bank aimed at understanding the complex system (penetrance modulators and mechanisms) involved in the development of BRCA-related cancers.

The aim of this report is to describe the scientific protocol of the e-BRAVE study.

## Methods

### Study design

About 100,000^45^ women with BRCA mutations live in Italy and presumably at least 50% of them are unaffected. The study aims:

- First, to create an Italian web-cohort of 2000 women carriers of *BRCA1/2* pathogenic variants. Within this cohort we aim to include at least 1000 carriers “unaffected” by any BRCA-related cancer with blood bank to investigate over time the role of potential penetrance modulators in the occurrence of primary BRCA-related cancers. In detail, the associations between genetic (polymorphisms), environmental modulators (including lifestyle and metabolic/hormonal/anthropometric/inflammatory factors), and the incidence of BC/OC/other BRCA-related cancers will be investigated;
- Second, to explore potential mechanisms by which the environmental modulators under study affect BRCA penetrance. Lipidomics, metabolomics, epigenetics (key miRNAs) and markers of microbiota (plasma SCFA) and dysbiosis (LPS) will be investigated.

The study was approved by the Ethics Committee of the Fondazione IRCCS Istituto Nazionale dei Tumori di Milano (INT) (approval number: INT30/24). The study plans to recruit the entire web-cohort in three years, and preliminary cancer incidence analyses on the unaffected carriers will be performed at the end of this period.

The e-BRAVE cohort represents the starting point of a potentially larger “open” digital platform to be followed-up for a longer period and to be implemented with additional BRCA people, and new data and analyses. The “open” digital platform development was shared with the EU4Health funded ELISAH project with the advantage to maximize funding and methodological synergie.

### Participants

Women carriers of *BRCA1/2* mutations following complete gene testing (sequencing of all coding regions and intron-exon boundaries, followed by Multiplex Ligation-dependent Probe Amplification assay for detection of large insertions/deletions), aged 18-75 years, and having tablet/mobile phone for internet access, are eligible. Since the focus of the e-BRAVE study is the incidence of BRCA-related cancers in unaffected women, the recruitment goal is to include in the study population at least 50% of women not affected by any BRCA-related cancer (~1000 women). Using a dedicated website, recruitment will be carried out for the three years of the study. A large multimedia campaign (television, radio, national and regional newspapers, posters, internet) is calling for volunteers by providing details on the study and its dedicated website, where volunteers can subscribe. A relay of information will be given, maintained and regularly updated on a large number of social networks, collaborating center websites, BRCA families’ associations and private firms. An additional invitation to participate is given by the professionals (geneticists, clinicians etc.) of the Institutions who are actively collaborating with the study, the IRCCS San Gerardo dei Tintori (Monza), the Istituto Tumori “Giovanni Paolo II” (Bari), The Center for Integrative Oncology-Fondazione Policlinico Universitario Gemelli IRCCS (Roma) and the Fondazione IRCCS Policlinico San Matteo (Pavia). All the participants in the web-cohort are requested to use the digital platform for data collection and activities. The unaffected women carriers are also required to provide a blood sample and undergo an anthropometric visit.

### Digital platform

An innovative digital platform, the LifeCharger (LC) SynCare Ecosystem, has been implemented to gather the web-cohort information, supporting women’s engagement, adherence to intervention plan, self-empowerment, flanked by activities tracking and monitoring. The interface is user-friendly, enabling seamless data collection, access to informative and educational content, and a secure channel for potential data sharing between participants and researchers. The LC SynCare Ecosystem architecture is based on three levels, declined into three different software applications:

1. The BRCApp, that is the end-user front-end and the user gate to the LC Ecosystem;
2. The Clinical Dashboard, that is the professional front-end and the software to monitor user activities and sends care/intervention plans. We are using the Clinical Dashboard to view and monitor patient/user data, and send feedback and action plans to users, closing the loop;
3. The backend, including the Data Analytics module, where data collected through the BRCApp are manipulated to be shown on the Clinical Dashboard. The data analytics module extracts user’s information by analyzing the data in form of “tags”. These tags are used to label the users during their journey inside the BRCApp. For example, if a woman has completed the profile with all the information, the Data Analytics module creates a tag that identifies this action as completed. The system becomes able to create clusters of users, applying the related journey including activities to performed in app (body parameters to be measured, questionnaires to be completed, and contents to read).

**Figure 1.**
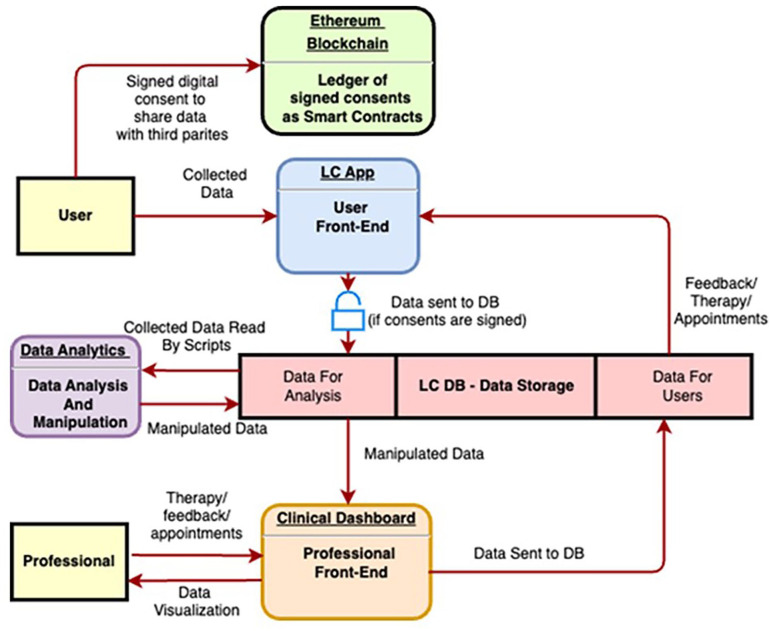
Platform dataflow.

Figure 1 describes the digital platform dataflow, starting from the user.

**Figure 2.**
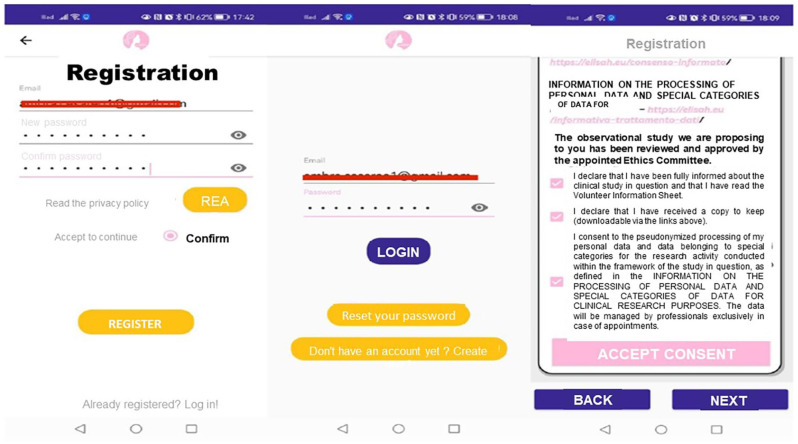
User registration and consent process in BRCApp.

Upon subscribing to the BRCApp (using email and password), women receive a confirmation email. At this point, they gain access to the BRCApp via the login section using their email and password, and sign the consent form (mandatory steps) (Figure 2).

**Figure 3.**
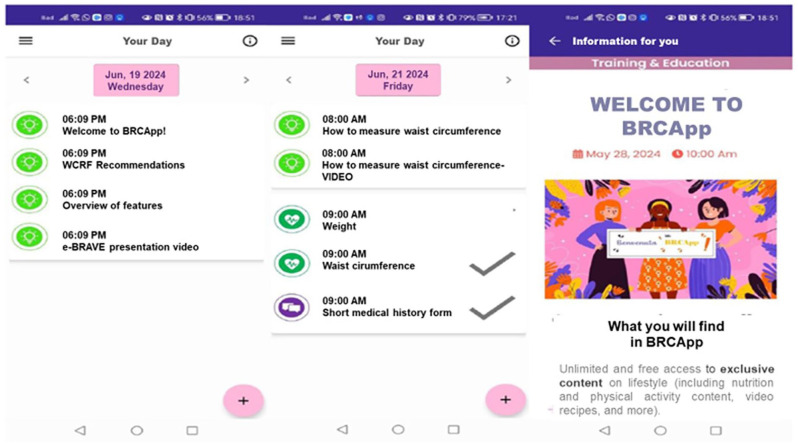
"Your Day" section: Daily activities and content overview.

Through the BRCApp HOME the women have access to different functionalities:

- “PROFILE”, that includes all the modules needed to gain basic information on users at the starting point;
- “YOUR DAY”, that lists all the activities scheduled for the day, allowing users to input requested measurements, fill in questionnaires, etc. (Figure 3);
- “APPOINTMENTS”, that allows users to view availability for booking appointments (anthropometric visit and blood examination), to view details about the reserved appointments (date, hour, location, additional information, etc.) and to cancel appointments;
- “ADHERENCE & REPORTS”, that provides an overview of user’s path, including adherence to treatment/intervention, trends of physiologic parameters, etc.;
- “DOCUMENTS”, that allows users to upload, manage and organize documents such as clinical reports, prescriptions, care plans, etc., by creating different folders and adding notes;
- “CONTENTS”, that collects all the informative and training materials received by the user, organized into categories (nutritional contents, video-recipes, video-exercises etc.) for easy access;
- “SERVICES”, that allows users to activate and manage services, such as webinar.

Once they have become a member, the women have to complete a short anamnestic form (including information on education and socio-economic condition), the short screener of adherence to Mediterranean diet (er-MEDAS), the Godin Shepard Leisure-Time Physical Activity questionnaire (GSL-TPAQ), and to book the clinical visit and blood examination according to a predefined list of appointments at the nearest collaborating center. Participants are required to provide a copy of their genetic test the day of the anthropometric visit. Reminder emails/in-app notifications are sent in order to obtain completed mandatory forms/blood samples and visit within three months from completing profile. Further questionnaires and activities are scheduled in the subsequent months following a pre-defined timeline and according to the season.

Women are also encouraged to regularly provide information on major health events. In case of reporting cancer or other significant health events (such as type II diabetes, stroke, myocardial infarction, ictus, etc.), medical records are requested to validate the information.

### Questionnaires

Participants, following a defined timeline, via BRCApp are asked to complete:

- The Energy-restricted Mediterranean Diet Adherence Screener (er-MEDAS),^46^ a short questionnaire on adherence to Mediterranean diet that captures the dimension of moderation of food consumption and energy restriction. Er-MEDAS consists of 17 items, 14 items on food frequency consumption and three on eating habits.
- The Godin Shepard Leisure-Time Physical Activity Questionnaire (GSL-TPAQ),^47^ to assess the amount of leisure time physical activity (PA) performed in the last seven days by including three questions about the frequency of PA (number of activities/week). Each question refers to PA of different intensity (one question on mild, one on moderate and one on strenuous PA) which corresponds to a specific Metabolic Equivalent of Task (MET) amount. Mild PA corresponds to 3 METs, moderate PA to 5 METs and Strenuous PA to 9 METs.
- The International Physical Activity Questionnaire (IPAQ)^48^ that identifies the total minutes over the last seven days spent on moderate PA, vigorous PA, walking, and inactivity. The questionnaire collects information on time (i.e. number of sessions and average time per session) spent walking, participating in moderate-intensity physical activity, participating in vigorous-intensity physical activity and sitting, on weekdays and weekend days. Questions regarding participation in moderate and vigorous physical activity are supplemented by concrete examples of activities commonly performed. Data from the questionnaire are summed within each item (i.e., vigorous-intensity activity, moderate-intensity activity, walking) to estimate the total amount of time spent on physical activity per week.
**-** The Reduced Morningness-Eveningness Questionnaire (r-MEQ)^49^ that consists of five multiple-choice items that investigate the chronotype, where lower scores indicate eveningness and higher scores morningness. Each response is assigned a score between 0 and 6.
- The Generalized Anxiety Disorder scale (GAD-7)^50^ that with seven items describes the most important diagnostic criteria according to DSM-IV-TR,^51^ namely Criterion A (fear and anxiety related to a series of events or activities), Criterion B (difficulties in controlling concerns) and Criterion C (anxiety and worry are accompanied by at least three additional symptoms such as restlessness, mild fatigue, difficulty concentrating, irritability, muscle tension and sleep problems). The GAD-7 asks how often people have suffered from the seven core symptoms of GAD within the last two weeks with the response options being “not at all,” “early days,” “more than half the days,” and “nearly every day,” scored as 0, 1, 2, and 3, respectively, with a total score ranging from 0 to 21.

### BRCApp contents

**Figure 4.**
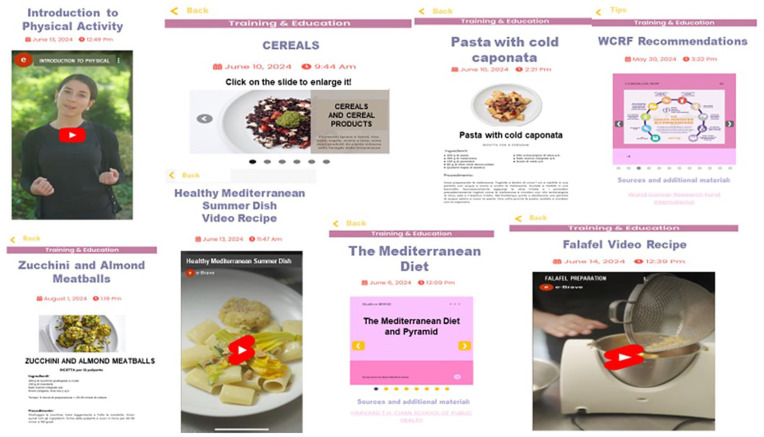
Lifestyle contents (recommendations, web kitchen/exercise courses, and recipes).

Through the BRCApp, participants have continuous free access to a wealth of content about lifestyle (recommendations, web kitchen/exercise courses, and recipes), wellness, study progress, potential risk/protective factors, and regulatory news (Figure 4). Lifestyle content (Online Supplementary Figure S1) includes textual content, recommendations, web kitchen/exercise courses, video-recipes/exercises and a calendar for participating in an informative webinar according to a pre-defined timeline. The textual content portions are short, easy to read, and initially on general topics (“The Mediterranean diet”, “The benefits of physical activity” etc.); after the first few months, as participants’ knowledge increases, the content becomes more focused (“The high-processed food” etc). The dietary content (mainly based on the Mediterranean principles/recipes) is created to improve women’ knowledge about diet as an important strategy for prevention, to gain greater awareness about food and nutrients, to help them choosing foods consciously and to control body weight. PA content is created to encourage women to reduce sedentary behavior, and to achieve and maintain regular participation in a moderate intensity physical activity for 210 minutes/week (30 minutes on average per day). PA contents include sessions, with detailed exercise descriptions and images and videos. The videos include simple and incremental exercises involving different muscle groups.

### Biobank and laboratory analyses

A volume of 25 mL of peripheral blood (in a fasting state) is collected in K_2_EDTA vacutainer tubes from the unaffected BRCA carriers included in the study. Starting from this volume, plasma and viable peripheral blood mononuclear cells (PBMCs) are separated and stored at -80°C for future immune profile analysis. Routine metabolic analyses (glucose, triglycerides, total cholesterol, HDL) are immediately performed. Further blood aliquots include:

- whole blood, for genetic analyses. Automated genomic DNA extraction from blood samples of participants will be done using the Maxwell RSC Whole Blood DNA Kit on a Maxwell Instrument (Promega). DNA will be quantified using a fluorimetric method (PicoGreen assay, Thermo Fisher Scientific). PLINK software^52^ will be used for controlling genotype quality and analyses. Haplotype phasing and imputation will be carried out using the TopMed or Michigan imputation servers.^53,54^
- plasma, for lipidomics/metabolomics, LPS, SCFA, and miRNAs analyses. The untargeted lipidomic/metabolomic profiling of plasma samples from the whole web-cohort will be performed according to the method developed by Segrado et al.^55^ LPS will be measured by Limulus Amebocyte Lysate assay using a commercial kit (HycultBiotech, Uden, The Netherlands). SCFA analysis will be performed according to the target method using Gas Chromatography-Flame Ionization Detection (GC-FID) and will be validated according to bioanalytical method validation.

MiRNA profiles will be processed and analyzed as previously reported.^56^ MiR-185, miR-498, miR-3910, miR-4423 and miR-4445 associated to BRCA and insulin/IGF pathways will be detected using fluorescent probes targeting miRNAs in Digital Droplet PCR assays (ddPCR, Bio-Rad);

- serum, for hormones/growth and inflammatory factors analyses. Immunometric assays will be performed with MAGPIX (Bio-Techne s.r.l, Milan, Italy) using the Luminex technique to measure Adiponectin, Leptin and a 3-plex inflammatory cytokine panel (IL6, IL10 and TNF). IGF-I and insulin will be assayed by commercial RIA kit purchased from DIAsource Immunoassy (Louvain-la Neuve, Belgium) and Immunotech (Prague, Czechoslovakia), respectively.
- buffy coat.

The storing of blood aliquots (at -80°C) is centralized at the biobank of the Nutrition Research and Metabolomics Laboratory (NuMeLab) of INT.

### Statistical analysis

Given the large volume and complexity of the data to be collected in this web-based cohort, we have developed a statistical analysis plan tailored to the two main study aims. First, we will examine the association between environmental and biological modulators (including lifestyle, hormonal/metabolic, anthropometric, and inflammatory factors) and the incidence of BRCA-related cancers (breast, ovarian, and others). Time-to-event data will be analyzed using Cox proportional hazards models to estimate hazard ratios and 95% confidence intervals. Both univariate and multivariable models will be implemented, adjusting for potential confounders such as age, menopausal status, parity, and other relevant factors. Since only women with mobile phone/tablet are included into the cohort, thus potentially introducing bias related to socio-economic status or digital literacy, we will examine whether outcomes vary across demographic subgroups and apply statistical adjustments for relevant covariates (e.g., socio-economic status), if necessary. Based on the incidence data of BRCA-related cancers^1,57^ and on the follow-up data from our MedDiet Trial^15^ we expect an overall incidence of ~3.7% year. We, therefore, estimate that approximately 50 new BRCA-related cancers will occur in three years, recruiting the major part of the cohort in the first 18 months. The expected number of incident cases will provide sufficient power to conduct robust multivariable analyses in line with event-per-variable (EPV) recommendations.

Second, we will explore potential mechanisms through which the environmental modulators affect cancer risk, focusing on biological intermediates such as lipidomic and metabolomic profiles, key microRNAs, short-chain fatty acids (SCFAs), and lipopolysaccharide (LPS) as a marker of dysbiosis. Cross-sectional analyses will be performed using linear regression for continuous variables and logistic regression for binary outcomes. In addition, the association between these biomarkers and cancer incidence will be analyzed using Cox models as described above.

## Results

The e-BRAVE study was launched at INT on 28 June 2024, and on the same day the digital platform/BRCApp opened for recruitment. Between July 2024 and January 2025, thanks to the recruitment activities, a total of 555 women carriers of BRCA mutations (of whom 293 unaffected) have downloaded the BRCApp by completing the registration process, and signed the informed consent. Among these, 497 women have completed all the data of the profile section and 346 already filled in the mandatory questionnaires (er-MEDAS and GSL-TPAQ).

Among the 293 unaffected BRCA women, 182 have booked the appointment for blood examination/anthropometric visit. Up to now, we have collected 120 blood samples in the e-BRAVE biological bank.

## Conclusions

Growing knowledge about *BRCA1*/*2*, alongside the use of BC/OC risk assessment/genetic testing has allowed thousands of women from high genetic risk families to learn their carrier status. Many of these women are young adults and often live much of their lives knowing their genetic risk of developing BC and/or OC. To date, non-surgical prevention options for women carriers are not yet firmly established^58^ and lifestyle recommendations are lacking. This web-based study represents the starting point for a potentially larger “open” cohort to be followed-up over time and implemented with additional BRCA people (including BRCA males), data (contact with clinicians/registry etc.) and contents. Success of this study will ensure the definition of further predictive risk models and comprehensive recommendations aimed at improving management and health status of families with BRCA mutations.

## Supplemental Material

sj-pdf-1-tmj-10.1177_03008916251353420 – Supplemental material for The e-BRAVE study: A prospective web-based cohort and biobank of women carriers of BRCA mutations Supplemental material, sj-pdf-1-tmj-10.1177_03008916251353420 for The e-BRAVE study: A prospective web-based cohort and biobank of women carriers of BRCA mutations by Andreina Oliverio, Carlotta Meli, Eleonora Bruno, Michela Bianchi, Giada Sassi, Elisabetta Venturelli, Ambra Cesareo, Claudio Pighini, Margherita Patruno, Maria Di Gennaro, Stefania Tommasi, Antonella Daniele, Silvia Schiavone, Letizia Galasso, Stefano Magno, Gianluca Franceschini, Alberta Ferrari, Robert Fruscio, Daniele Morelli, Claudia Chiodoni, Siranoush Manoukian and Patrizia Pasanisi in Tumori Journal
