# Supplementary material for: The e-BRAVE study: A prospective web-based cohort and biobank of women carriers of BRCA mutations
Source: Tumori. 2025 Aug 9;111(5):390–9. doi: 10.1177/03008916251353420 (PMC12476487; doi:10.1177/03008916251353420)
Supplement: sj-pdf-1-tmj-10.1177_03008916251353420 – Supplemental material for The e-BRAVE study: A prospective web-based cohort and biobank of women carriers of BRCA mutations [file sj-pdf-1-tmj-10.1177_03008916251353420.pdf]

Supplementary materials

Figure S1- Examples of lifestyle content in BRCApp

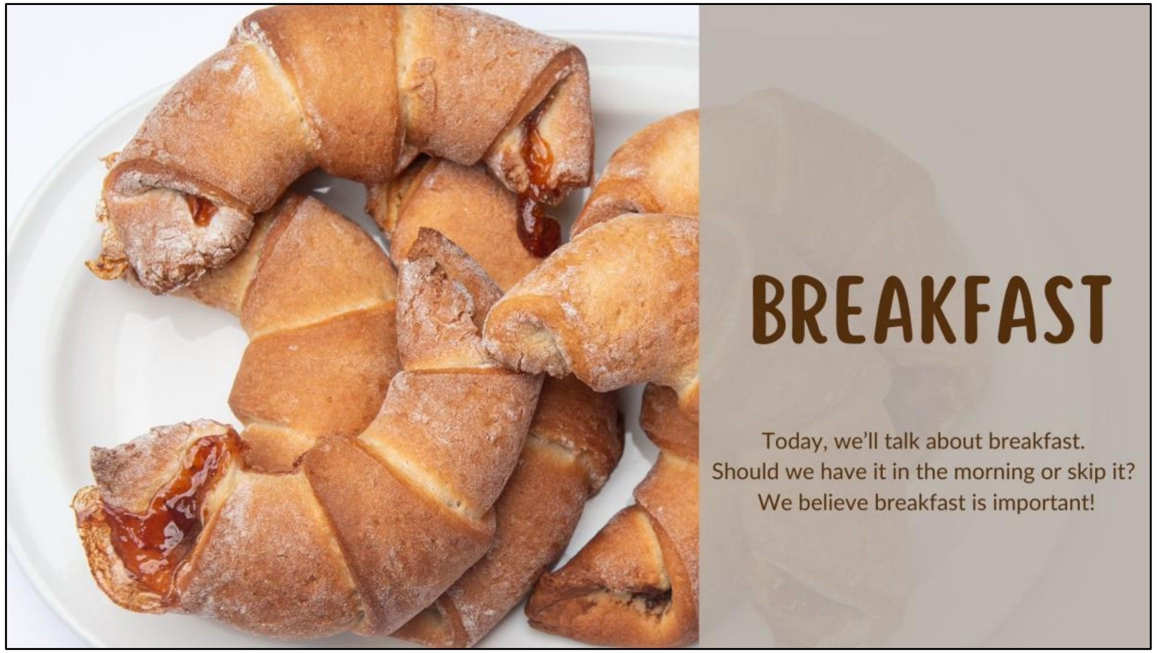

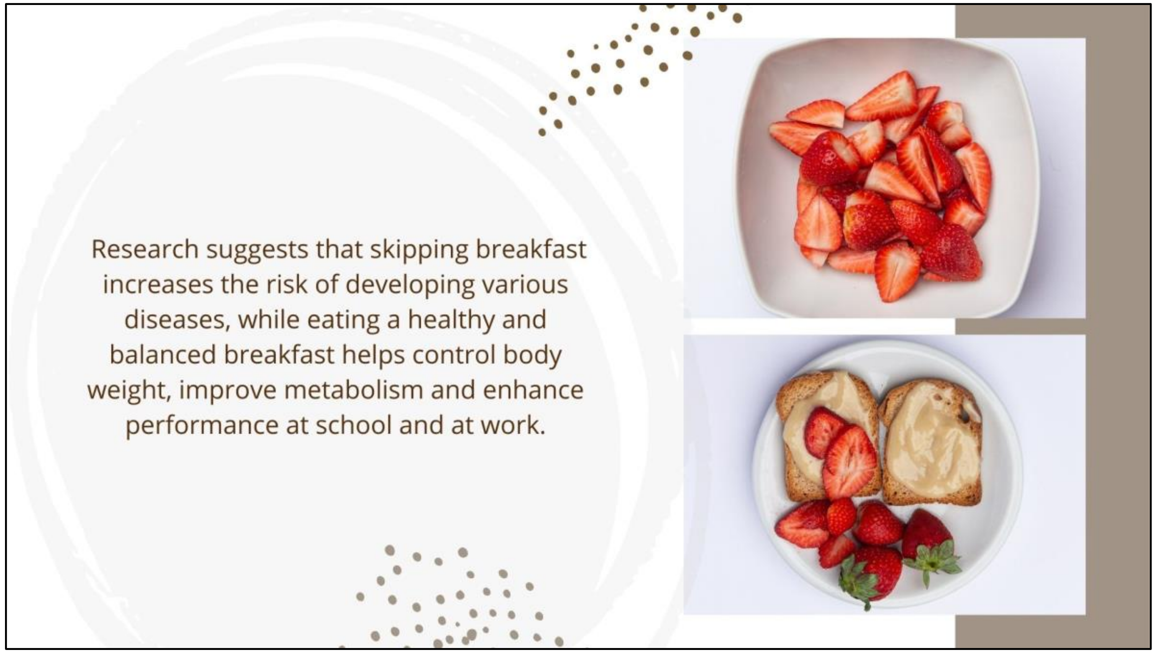

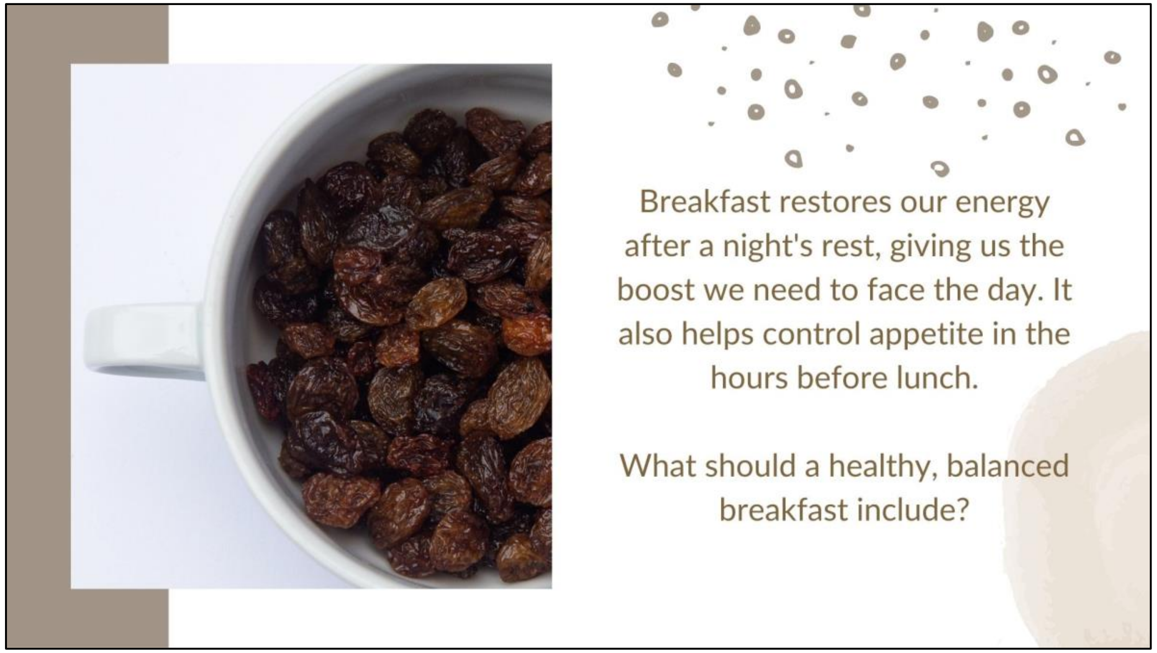

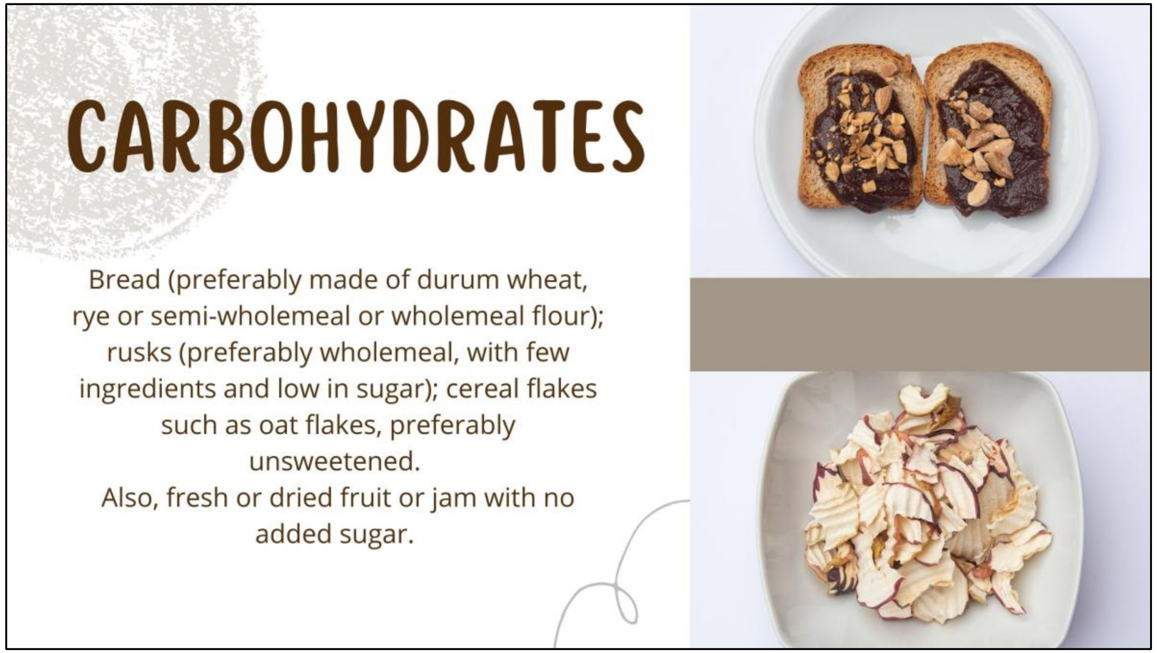

PROTEINS

Yogurt (white, Greek or plant-based), but also kefir or skyr; milk, skimmed if possible, no more than 125 ml daily.

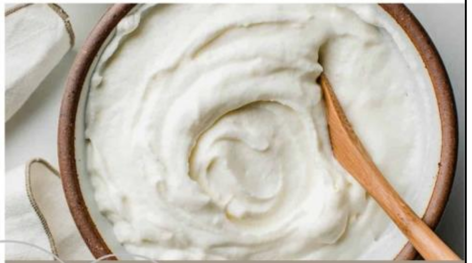

HEALTHY FATS

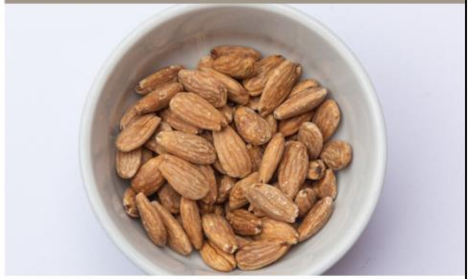

Nuts, 100% nut creams such as almond or hazelnut butter; oily seeds to add to cereals and yogurt; extra virgin olive oil (with toast and tomato for a savoury breakfast).

If you like starting the day with a hot drink like coffee, barley coffee or tea, it's best not to add sugar.

If you're looking for an alternative to cow's milk, try plant-based substitutes such as soy milk (nutritionally closest to cow's milk), cereal drinks (oats, kamut, spelt, rice) or nut drinks (almond, walnut, hazelnut).

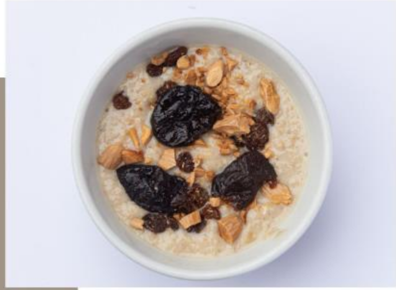

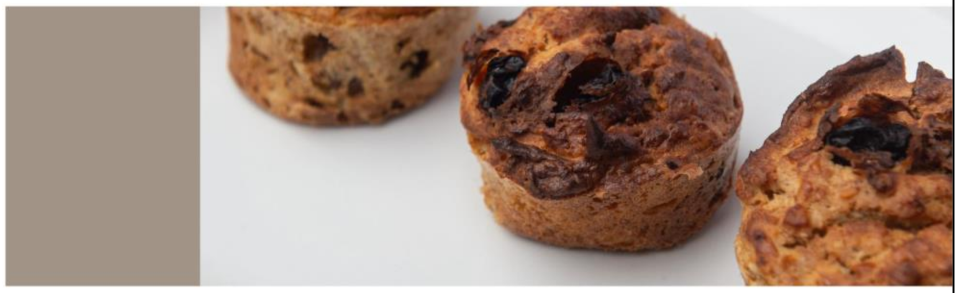

And now it's your turn to try out our video recipes!

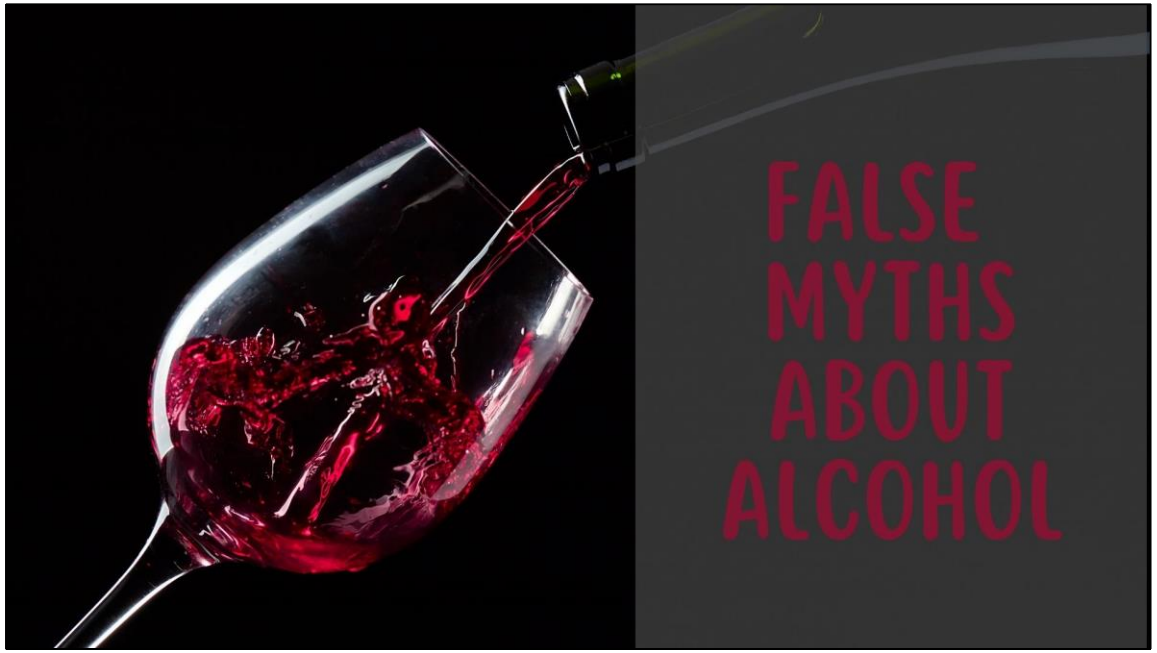

FALSE MYTHS ABOUT ALCOHOL

Alcoholic beverages, especially beer and wine, are widely consumed in many European countries.

An **alcoholic beverage** is defined as any beverage that contains alcohol (ethanol) in quantities starting from 1.2 ml per 100 ml.

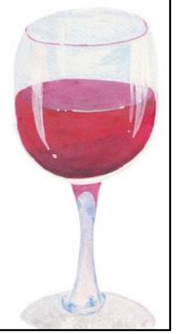

The labels of alcoholic beverages display the **alcohol content**, that is, the number of millilitres of pure alcohol in 100 ml, expressed as *% vol*.

The average alcohol content of alcoholic beverages varies widely.

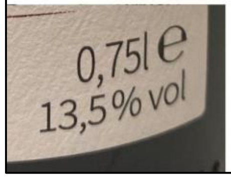

The table below lists some examples of the most commonly consumed types.

TYPE OF ALCOHOLIC BEVERAGE	ALCOHOL CONTENT (% vol)
Non-alcoholic beer	0.5
Light-coloured beer	5
Double malt beer	8
Table wine (red, white)	Tra 11 e 13
Sweet white dessert wine, sparkling wine	Tra 12.5 e 16
Aperitifs	22
Bitter	Tra 23 e 35
Dessert liqueurs	Tra 31 e 35
Vodka, gin, rum	37.5
Grappa, whisky	40

FALSE BELIEFS ABOUT ALCOHOL

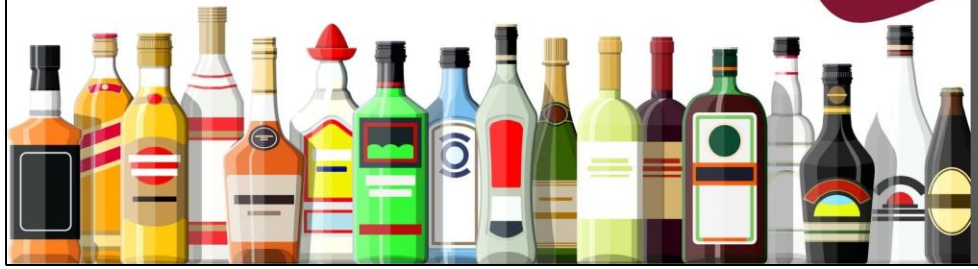

Alcohol aids digestion.
False! It actually causes inflammation of the gastric mucosa with reduced production of digestive enzymes and mucus, along with hypersecretion of gastric acid and delayed emptying of the stomach.

Alcoholic beverages quench thirst.
False! They actually dehydrate and increase water loss through urine and sweat.

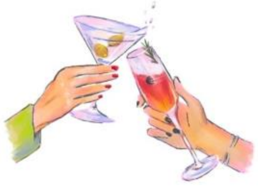

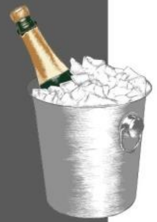

Alcohol makes you warm.
False! It actually causes vasodilation, giving a momentary and deceptive sensation of warmth.

Alcohol gives strength.
False! In reality, it merely reduces the sensation of fatigue and pain.

Alcohol from beer and wine is less harmful.
False! Though it is true that beer and wine have a lower alcohol content than spirits, a 33 cc glass of beer contains the same amount of alcohol as a 15 cc glass of wine or a 4 cc glass of spirits..

Alcohol is an aphrodisiac.
False! In fact, especially at high doses it has a depressive effect on the central nervous system and negatively impacts sexual performance.

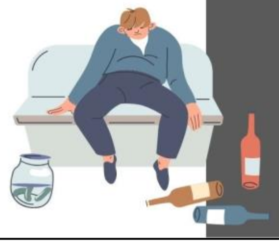

Non-alcoholic beers are alcohol-free.
False! They do contain a small amount of alcohol (as shown in the table), but it's not mandatory to indicate this on the label.

Beer helps replenish fluids and electrolytes after physical exertion.
False! Although beer may seem like a good post-workout supplement because of its carbohydrate and mineral supply, it slows down rehydration and protein synthesis due to its alcohol content, thereby delaying recovery.

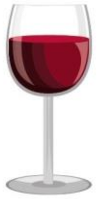

Wine 'makes good blood'.
False! In reality, ethanol is a toxic substance that reaches various organs through the bloodstream, with possible harmful effects.

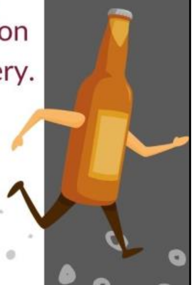

Drinking a lot, but only occasionally, is not harmful.

False! Occasional excessive alcohol consumption, also known as binge drinking, can lower a person's risk perception and may lead to acute alcohol poisoning, which in some cases will require the intervention of qualified medical personnel.

Beer increases milk production in pregnant women.

False! Although some studies have suggested that beer increases the production of prolactin (a hormone that stimulates milk production after childbirth), drinking beer during lactation was found to be associated with lower milk intake by infants. Moreover, the passage of alcohol through breast milk can have harmful effects on the baby.

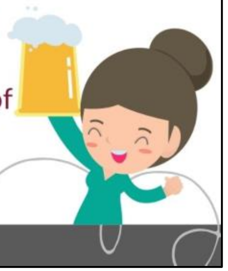

e-BRAVE study

Mediterranean diet and pyramid

Exploring the Mediterranean diet

'Mediterranean diet' refers to the typical alimentary style of the countries of the Mediterranean region.

It is characterised by the consumption of lightly refined grains, pulses, nuts, vegetables and fruits typical of the Mediterranean, with extra virgin olive oil as the main source of fat.

Many studies have shown that following a Mediterranean diet reduces the risk of cardiovascular disease, helps prevent diabetes, protects against certain types of cancer, promotes the maintenance of a healthy body weight and improves cognitive function.

The Mediterranean diet can be represented as a pyramid to help you distinguish which foods to favour (those at the pyramid's base), which to consume in moderation (those in the middle) and which to consume with caution (those at the top).

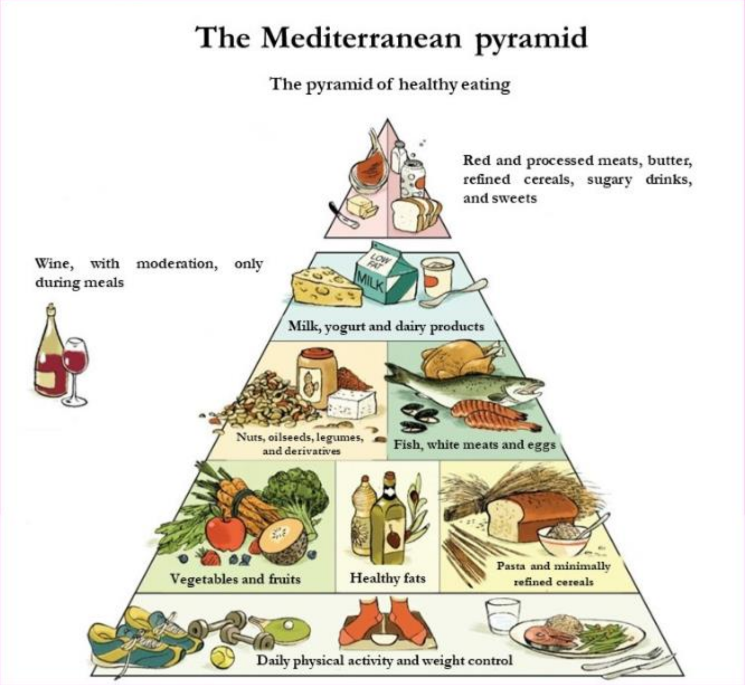

→

The base of the Mediterranean Diet Pyramid is completely plant-based. These foods make up the daily foundation of the diet:

- **Grains and grain products (preferably wholegrain)** such as pasta, rice, other grains, bread, etc.), to be eaten daily at every meal;
- **Pulses** (beans, chickpeas, lentils, etc., both dried and fresh), to be eaten daily or at least 2 or 3 times a week;
- **Vegetables and fruits**, at least 5 servings per day (about 500-600 g in all);
- **Nuts** (almonds, walnuts, hazelnuts, etc.), 1 serving (about 30 g), perhaps as a snack, at least 3 times a week.

Moving up to the higher levels of the pyramid, we find foods that should be consumed somewhat less frequently:

- **Fish**, to be eaten 3 times a week (serving size 150 g);
- **White meat**, to be eate[illegible]wice a week (serving size 100-120 g);
- **Eggs**, to be eaten twice a week
- **Milk**, preferably low-fat, or (preferably) fermented milk derivatives such as yogurt, 1 serving per day, about 125 ml (equal to a small glass of semi-skimmed milk or a small pot of yogurt);
- **Cheese**, especially if high in fat, to be eaten no more than twice a week (serving size 100 g for fresh cheeses and 50 g for mature cheeses).

At the top of the pyramid we find foods to be consumed occasionally, such as:

- **Red meat and preserved meats** (e.g., cold cuts and sausages), to be eaten no more than once a week (serving size 100 g for red meat and 50 g for preserved meats);
- **Sweets** (maximum 1-2 times a week), preferably home-made.

Extra tips:

- **Drinks,** prefer water and avoid fizzy and sugary drinks;
- **Condiments,** favour extra virgin olive oil as the main condiment (2-4 tablespoons per day). Limit butter, margarine and cream;
- Even though **WINE** is part of the Mediterranean diet, we recommend moderate intake. For cancer prevention, the advice is to refrain from consuming alcoholic beverages altogether. If you do drink alcohol, it is recommended that you limit yourself to a maximum of 1 glass of wine per day (125 ml) during meals only. The amount of alcohol in a glass of wine is approximately equal to that in a can of beer or a shot of spirit or liqueur.

e-BRAVE study

Seasonality

What is it seasonality?

Fruits and vegetables play a crucial role in the Mediterranean diet, providing useful nutritional benefits for health.
However, it is important to learn how and when to put these foods on the table.

Although a wide variety of fruits and vegetables can be found in supermarkets all year round, we must remember that these foods follow a seasonal cycle.

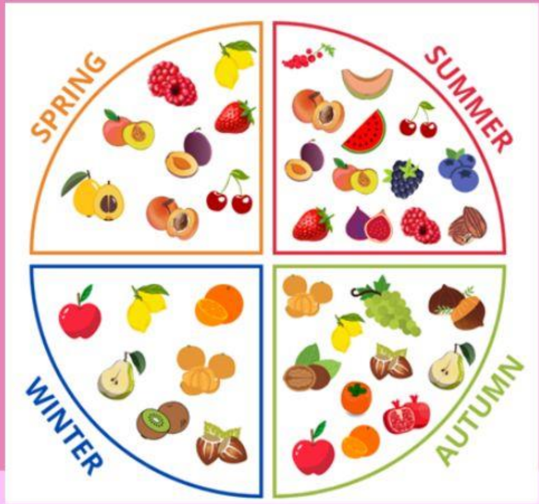

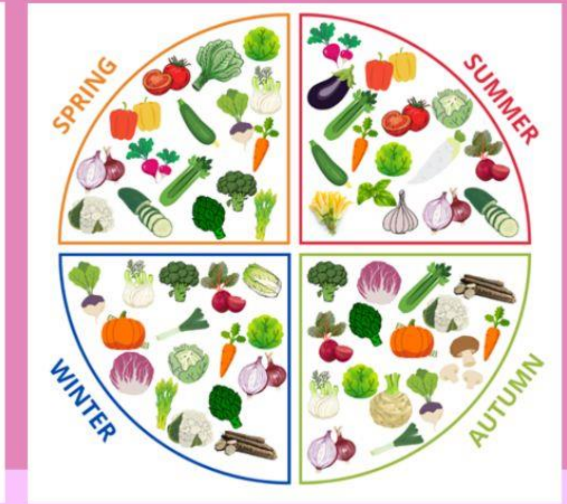

→

Observing the seasonality of fruit and vegetables means:

- Maximising taste and nutritional quality;
- Contributing to environmental sustainability and resource conservation.

Nature works very well for us and every season gives us what we need!

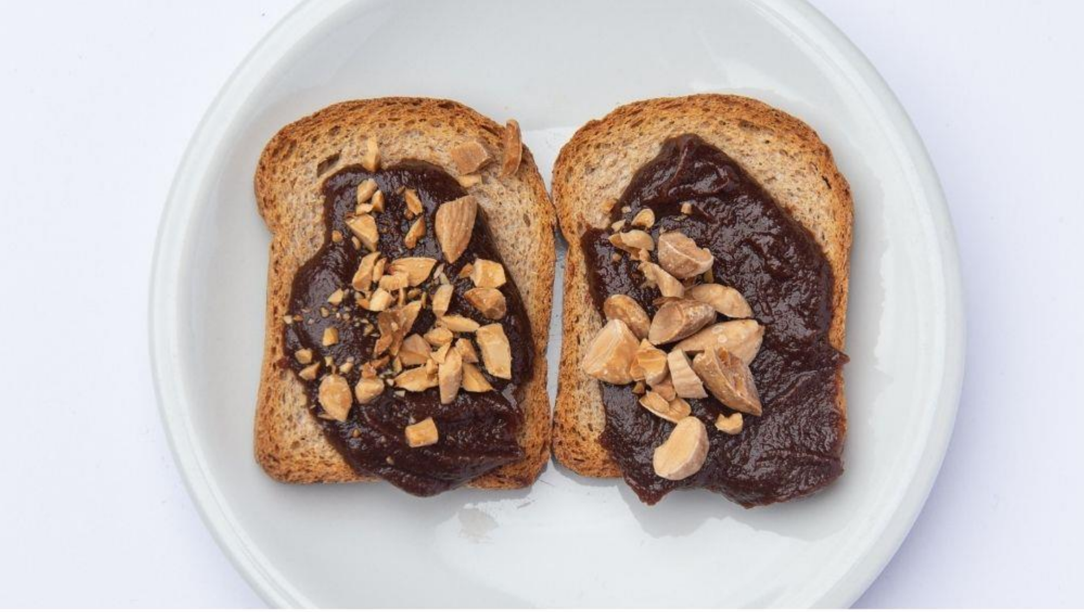

Hazelnut cream spread

RECIPE FOR 4 PEOPLE

Ingredients:

- 2 tablespoons hazelnut cream 100%
- 1 tablespoon unsweetened cocoa powder
- 1 tablespoon rice malt
- Rice milk to taste

Method:

Mix 2 tablespoons of hazelnut cream, 1 tablespoon unsweetened cocoa powder and 1 tablespoon of rice malt with the rice milk until you get the desired consistency.
Enjoy it for breakfast on a rice cake, slice of wholemeal bread or wholemeal rusk. Can also be used as a pancake filling or as an accompaniment to cooked apples.

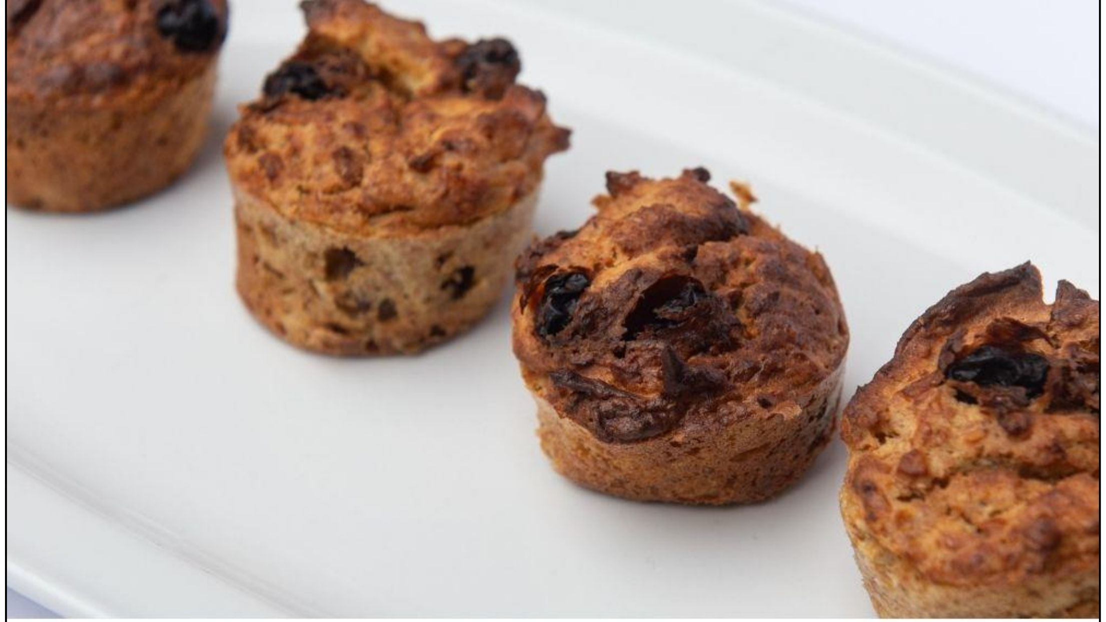

Muffins

RECIPE FOR 4 PEOPLE

Ingredients:

- 200 g semi-wholemeal flour
- 50 ml corn oil
- 1-2 grated apples
- 100 g raisins
- 50 ml apple juice
- 50 g chopped hazelnuts
- Grated lemon zest
- 1 vial of vanilla flavoring
- A pinch unrefined sea salt
- ¾ sachet cream of tartar

Method:

Combine the semi-wholemeal flour and cream of tartar in a bowl with a pinch of unrefined sea salt. Mix the apple juice and corn oil. Add the wet ingredients to the dry and stir together to make a dough. Add the apples, raisins, chopped hazelnuts, vanilla and lemon zest and pour the mixture into the moulds. Bake in a pre-heated oven for 45 minutes at 180°C. Once cooked and golden, they're ready to be enjoyed!

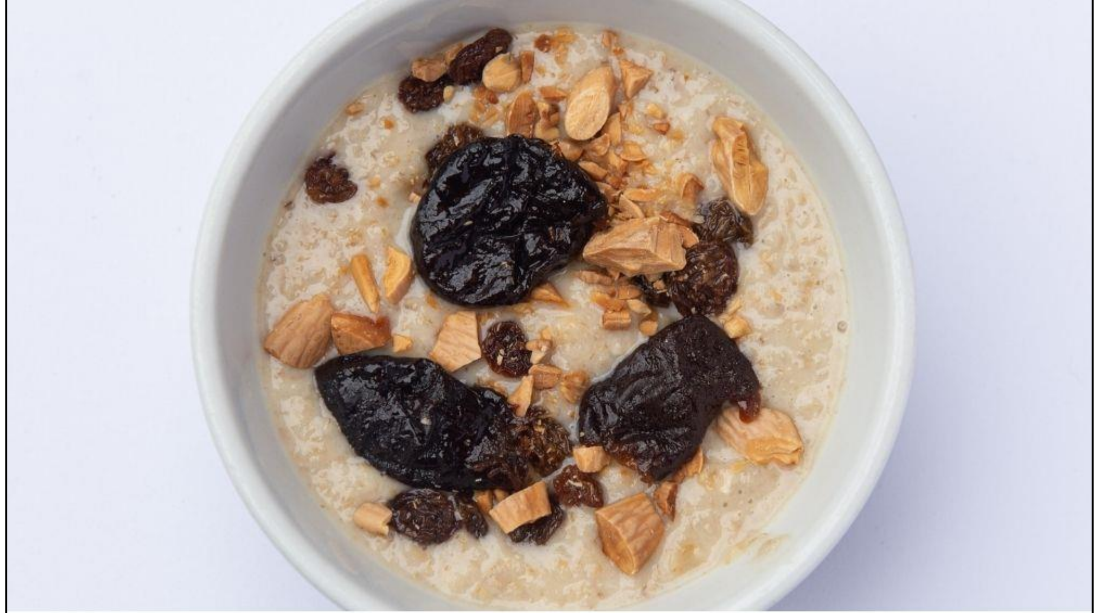

Porridge

RECIPE FOR 4 PEOPLE

Ingredients:

- 1 cup oat or barley flakes
- 3-4 cups water, rice milk, oat milk or apple juice
- A pinch unrefined sea salt
- 1 teaspoon raisins, dried apricots or prunes

Method:

In a saucepan, combine the oat flakes, oat milk and a pinch of salt. Bring to the boil and simmer over a low heat for 15-20 minutes until the porridge reaches a creamy consistency. Start stirring when it's almost ready to stop it from sticking to the bottom of the pan. Serve the porridge in bowls and top with pieces of apple, raisins and roughly chopped toasted almonds to add crunch. You can use dried apricots or prunes instead of raisins if you prefer.

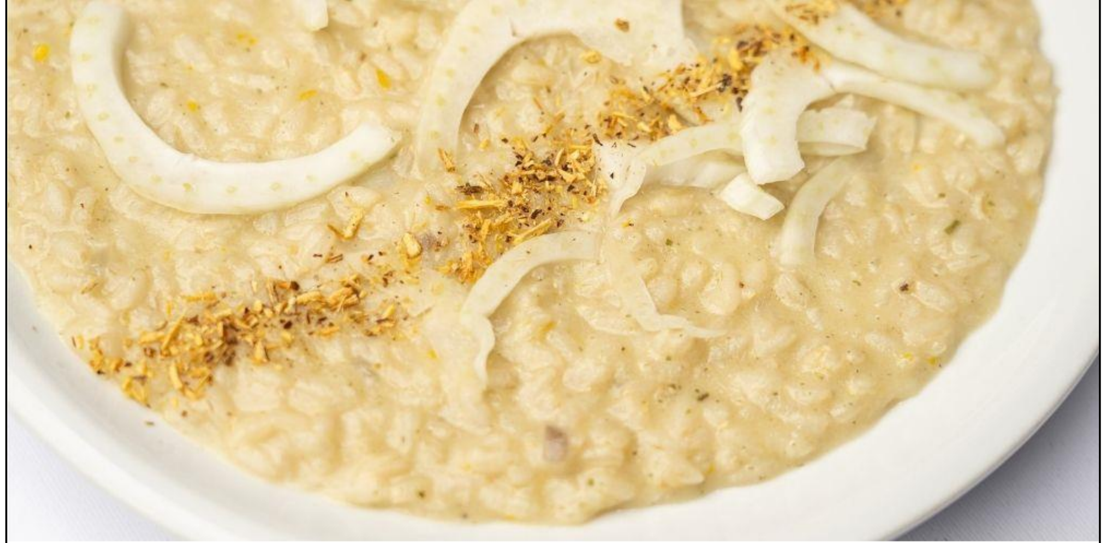

Fennel, orange, and liquorice risotto

RECIPE FOR 4 PEOPLE

Ingredients:

- 320 g semi-milled brown rice
- Vegetable stock to taste
- 200 g fennel
- 1 untreated orange
- 1 tablespoon powdered liquorice
- 50 g white onion
- Extra virgin olive oil to taste
- Nutritional yeast flakes
- Unrefined sea salt to taste
- Black pepper to taste

Method:

Heat a dash of olive oil in a casserole dish and fry the finely chopped onion. Add the rice and toast briefly before adding a ladle of vegetable stock and allowing to reduce. Carry on cooking by gradually ladling in more stock. Meanwhile, clean the fennel, slice it thinly and cook in a frying pan with a little olive oil until soft. Once cooked, blend the fennel to a paste. When the rice is almost cooked, add the fennel paste and, once it has finished cooking, give the risotto a creamy consistency by adding a drizzle of olive oil, the yeast flakes, a pinch of salt, a little pepper, the grated orange zest and the powdered liquorice. Serve the risotto garnished with a few slices of raw fennel, orange zest and a pinch of powdered liquorice.
